# Tumor‐associated macrophages: An important player in breast cancer progression

**DOI:** 10.1111/1759-7714.14268

**Published:** 2021-12-15

**Authors:** Xinqun Huang, Jingsong Cao, Xuyu Zu

**Affiliations:** ^1^ Institute of Clinical Medicine, The First Affiliated Hospital of University of South China Hengyang China

**Keywords:** breast cancer, tumor immune microenvironment, tumor‐associated macrophages (TAMs)

## Abstract

Breast cancer is the most common form of malignant tumor in females, accounting for the second highest mortality among cancer patients. In the breast tumor microenvironment, tumor‐associated macrophages (TAMs) are the most abundant immune cells, which regulate the progression of breast cancer. During breast cancer tumorigenesis and progression, TAMs support breast tumor growth by promoting angiogenesis and cancer cell metastasis, inducing cancer stemness, regulating energy metabolism, and supporting immune system suppression. TAMs exhibit a high degree of cellular plasticity. Repolarizing tumor‐related macrophages into M1 macrophages can promote tumor regression. This study reviews the role and mechanism of action of TAMs in the development of breast cancer and establishes TAMs as effective targets for breast cancer treatment.

## Graphical abstract

TAMs support the progression of breast cancer by promoting tumor cell invasion and metastasis, angiogenesis, cancer stemness, regulating energy metabolism, and modulating T cell activity.

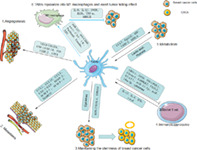

## INTRODUCTION

The global cancer incidence and prevalence statistics of 2020 indicated that breast cancer has surpassed lung cancer to become the most common form of cancer prevalent in women today.^1^ Histological classification of breast cancer based on the expression of estrogen receptor (ER), progesterone receptor (PR), and/or human epidermal growth factor receptor‐2 (HER2) has been established as the gold standard of cancer diagnosis which is clinically used to classify breast cancer as luminal A, luminal B, HER2‐positive and basal‐like triple negative breast cancer (BL/TNBC).^2^ Clinical efforts to characterize the features of the breast tumor microenvironment (TME) have confirmed the presence of tumor cells and the active recruitment of host immune cells such as tumor‐associated macrophages (TAMs), T cells, natural killer (NK) cells, B cells, granulocytes, plasma cells, and basophils, respectively.^3^ TAMs are prominent components of the TME, comprising over 50% of the total infiltrating immune cells in some cases, and can affect the progression of breast cancer through diverse mechanisms^4^, ^5^ Macrophages as a heterogeneous cell population are differentiated into two functionally distinct subtypes which respond to different environmental factor‐based stimuli to form classical activated M1 macrophages or alternatively activated M2 macrophages, respectively.^6^, ^7^

**FIGURE 1:**
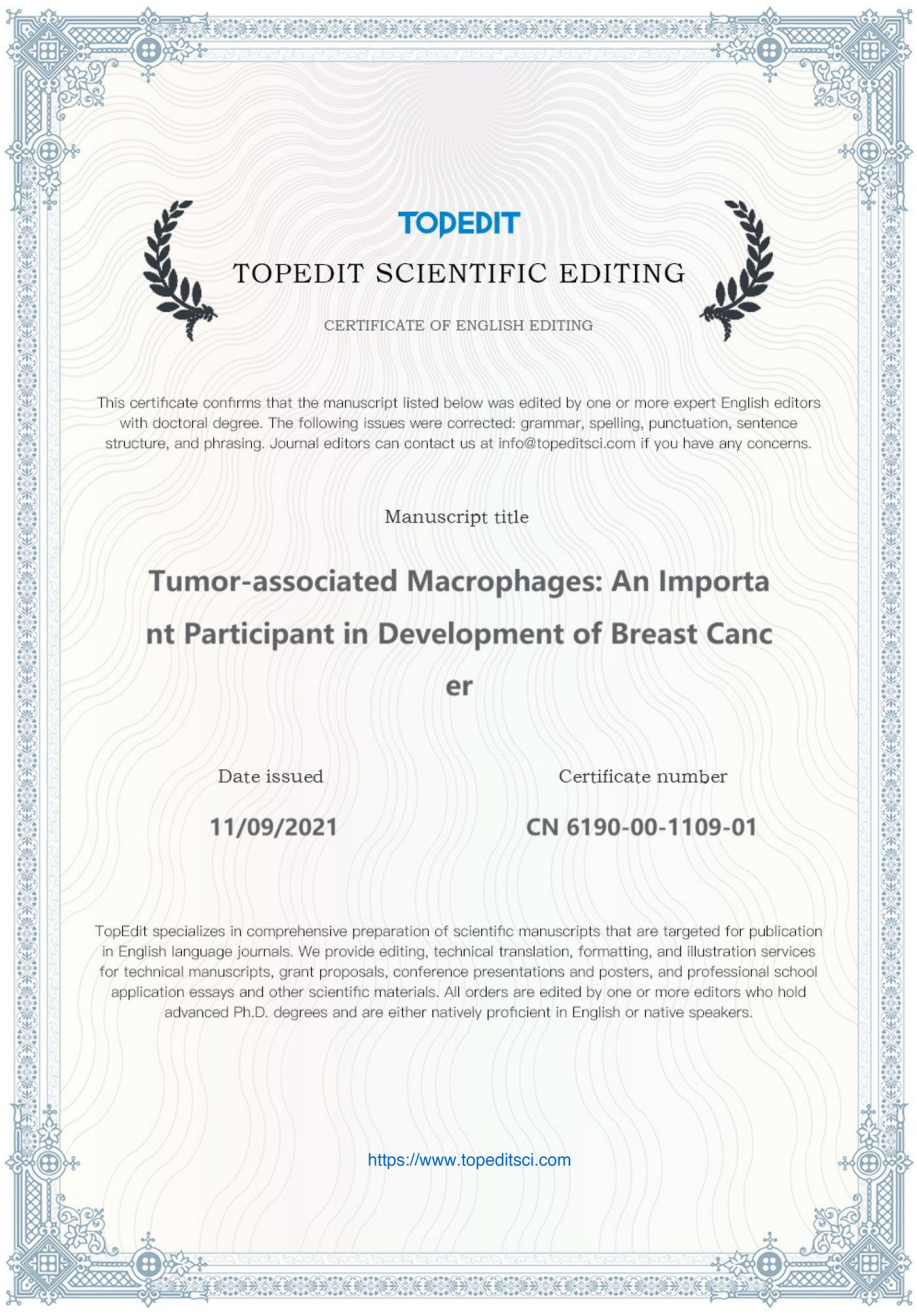
TAM‐associated mechanisms which promote the development of breast cancer

Traditionally, M1 macrophages exert tumor‐killing functions via cancer cell recognition and phagocytosis accompanied with production of proinflammatory cytokine molecules such as interferon γ (IFN‐γ) and interleukin‐12 (IL‐12).^8^, ^9^, ^10^ During tumor progression, the number of M2 macrophages increase and they become the dominant type of TAM in the TME. M2 macrophages are generally regarded as “tumor promotors”, which support the progression of breast cancer by promoting tumor cell invasion and metastasis, angiogenesis, cancer stemness, regulating energy metabolism, and supporting immune system evasion^.^ ^5^, ^11^, ^12^, ^13^, ^14^ TAM‐based infiltration in the primary tumor has been associated with inferior patient prognoses and treatment outcomes.^5^, ^11^, ^12^, ^13^, ^14^ In this review, we summarize the functional aspects of TAMs in the development of breast cancer as shown in Figure 1 which may be utilized in breast cancer diagnosis and prevention.

## BREAST CANCER ANGIOGENESIS

Angiogenesis involves the formation of new blood vessels which support tumor growth and development. TAMs act as important players in angiogenesis by closely associating themselves with high‐density vascular networks formed in breast cancer. In breast cancer TME, TAMs are an important source of vascular endothelial growth factor (VEGF).^15^, ^16^ The interactions of VEGF with vascular endothelial growth factor receptors (VEGFRs) triggers angiogenesis in breast cancer. Therefore, inhibiting potential VEGF/VEGFR interactions can significantly block angiogenesis and tumor metastasis.^17^, ^18^ The macrophage colony‐stimulating factor or colony‐stimulating factor 1 (CSF‐1) drives the recruitment and the differentiation of macrophages towards a M2 phenotype. During neoplasm development in the mammary gland, the application of colony‐stimulating factor 1 receptor (CSF1‐R) inhibitors can deplete TAMs to effectively inhibit metastasis, angiogenesis and reduce the invasiveness of the tumor.^19^

Hypoxia is a hallmark of the TME that promotes angiogenesis and leads to efficient recruitment of macrophages.^20^ The hypoxic environment activates macrophages to transform into TAMs stimulating the upregulation of hypoxia‐inducible factors (HIFs) in TAMs, which act as transcriptional activators of VEGF.^21^, ^22^VEGF facilitates hypoxic microenvironment‐based angiogenesis which supports oxygen and nutrient delivery to the tumor, promoting its growth.^21^, ^22^ Early evidence of the role of HIF signaling has been correlated with angiogenesis, inhibition of the HIF‐1α signaling which impedes angiogenesis and tumor growth. Interestingly, inhibition of the HIF‐2α signaling leads to the formation of highly disordered blood vessels and aggravation of the hypoxic condition in the TME.^20^ Additionally, breast cancer cells in the hypoxic TME upregulate the expression of activating transcription factor 4 (ATF4), a member of the ATF/cAMP response element‐binding protein (CREB) family, which has been reported to be related to the recruitment of macrophages and promotion of angiogenesis which indirectly support tumor growth.^23^ Thus, TAMs increase the malignancy of tumors by promoting angiogenesis.

## BREAST CANCER CELL METASTASIS

Metastasis is the primary cause of death in breast cancer patients. TAMs play a key role in promoting metastasis and invasion in breast cancer. Targeting TAMs has previously been suggested as a potential therapeutic strategy for the treatment of metastatic breast cancer.^5^, ^24^TAMs facilitate tumor metastasis by chemokine (C‐C motif) ligand2 (CCL2), CCL5, and CCL18, respectively. The functional mechanism of CCL2 involves the promotion of metastasis in breast cancer cells to bone and lung tissue. The CCL2‐expressing breast tumor cells recruit C‐C motif chemokine receptor 2+ (CCR2+) macrophages to accumulate in the lung and regulate osteoclast differentiation in the bone, playing a significant role in premetastatic niche formation by cancer cell colonization. Thus, inhibition of CCL2‐CCR2 may effectively inhibit tumor metastasis.^25^, ^26^ Breast cancer cells that secrete CCL5 act on mononuclear macrophages towards TAMs which can promote tumor migration and invasion.^27^ CCL18 is abundantly released by TAMs, and its expression in TME is associated with tumor metastasis and decreased patient survival. The PYK2 N‐terminal domain‐interacting receptor 1 (PITPNM3), which is the functional receptor of CCL18, inhibits the metastatic and invasive effects exerted by CCL18.^28^ Nie and colleagues reported the existence of positive feedback loops of CCL5‐CCR5 and CCL18‐PIPTNM3 between malignant phyllodes tumors (PT) of the breast and TAMs, while assisting in maintaining TAM phenotype as well as PT aggressiveness. Their study reported the use of CCR5 inhibitor and CCL18 monoclonal antibody to double‐block the CCL5‐CCR5 and CCL18‐PIPTNM3 pathways, which led to significant suppression of tumor metastasis.^29^ TAMs secrete cellular cytokines and surface receptors which are important factors promoting breast cancer metastasis. High epidermal growth factor (EGF) expression in TAMs activates epidermal growth factor receptors (EGFRs) in the cancer cells which in turn promotes metastasis and CSF‐1 secretion. The CSF‐1 recruits and activates TAMs to further secrete EGF, which suggests the existence of an EGF/CSF‐1 positive feedback loop between TAMs and cancer cells. EGF induces the infiltration of breast cancer cells into the blood vessels, leading blood vessel metastasis.^30^ A group of matrix‐metalloproteinases (MMPs), such as MMP2, MMP7 and MMP9 are secreted by TAMs, which have been demonstrated to be involved in the degradation matrix components of the TME, promoting the metastasis of tumor cells and the formation of the metastatic microenvironment.^15^ High expression of the scavenger receptor named macrophage receptor with collagenous structure (MARCO) by suppressive TAMs promotes tumor growth and metastasis. MARCO is closely associated with metastasis driving gene signatures for epithelial‐mesenchymal‐transition (EMT), and targeted blocking of MARCO expression can effectively inhibit tumor metastasis.^31^

## TAMS PROMOTE BREAST CANCER CELL STEMNESS

TME consists of a large number of immunosuppressive cells (mainly TAMs). There is evidence to support that TAMs induce and maintain cancer stem cells (CSCs), thereby promoting tumorigenesis, proliferation, and self‐renewal.^5^, ^32^, ^33^ There is a vast body of published literature that supports the involvement of the various TAM‐based cytokines in the generation of breast CSCs. It was earlier thought that classical “M1” activation exerts antitumor effects via proinflammatory cytokines which prevent tumor progress. A recent study by Guo and colleagues showed that the proinflammatory effects of M1 can also trigger the expansion and self‐renewal of CSCs. Coculture of breast cancer cells with M1 macrophages induced the formation of aldehyde dehydrogenase 1+ (ALDH1+) breast CSCs through inflammatory cytokine activation of the Lin‐28B‐let‐7‐ HMGA2 pathway, and these breast CSCs were highly drug‐resistant with elevated spheroid forming capability. Their study also suggested that the M1 phenotype repolarized into the M2 phenotype to maintain a high population of ALDH1+ breast CSCs.^34^ The IL‐6 from TAMs can promote the transformation of human and mouse nonstem cancer cells (NSCC) into CSCs by activating the JAK/STAT pathway to enhance the self‐renewal and tumorigenic capacity of CSCs.^35^, ^36^ Immune‐suppressing M2‐like macrophages in inflammatory breast cancer (IBC) have been found to secrete high levels of IL‐8 and growth‐regulated oncogene (GRO) chemokines which activate the STAT3 pathway, and are the main driving force for the formation of the CSCs.^37^

Additionally, TAMs also promote breast cancer cell stemness by upregulating the expression of the SRY‐related HMG‐box (SOX) family of transcription factors (TFs) and surface receptors. The EGF secreted by the TAMs activates the EGFR/ STAT3/SOX‐2 paracrine pathway in the breast cancer, resulting in increased SOX‐2 expression, which in turn enhances the CSC phenotype in the tumor cells.^38^ The existing body of research on SOX‐2, OCT‐4 and NANOG suggests that early‐stage breast tumors exhibit SOX‐2 expression, with no expression of OCT‐4 and NANOG. Overexpression of SOX‐2 increased the spheroid‐forming ability and self‐renewal in CSCs,^39^ suggesting that SOX‐2 is a key molecule regulating the formation of CSCs in early breast cancer. Transforming growth factor‐β (TGF‐β) upregulates SOX‐4 expression during the EMT process, and SOX4 directly enhances the expression of histone methyltransferase EZH2. Overexpression of EZH2 is essential in stem cell self‐renewal and the expansion of CSCs in breast cancer.^40^, ^41^The ephrin type‐A receptor 4 (EPHA4) protein on the surface of TAMs is upregulated during EMT and binds directly to the receptor on cancer cells, which activates the NF‐kB pathway in cancer cells to facilitate the maintenance of homeostasis in the CSCs.^10^

## TAMS MODULATE T CELL ACTIVITY TO INDUCE IMMUNOSUPPRESSIVE MICROENVIRONMENT IN BREAST CANCER

The immunomodulatory function of TAMs is a major mechanism of cancer disease progression and the main area of focus here is the regulation of the tumor‐killing function of effector T cells.^5^ TAMs regulate arginine metabolism as an important way to suppress T cell function. The expression level of arginase‐1 (ARG‐1), a molecular marker of M2 macrophages, has been reported to be significantly higher in breast cancer patients compared with healthy controls. The level of L‐arginine decreases to suppress the function of effector T cells in condition of ARG‐1 hydrolyzes L‐arginine.^42^ In addition to ARG‐1, nitric oxide synthase (iNOS), a molecular marker of M1 macrophages, metabolizes L‐arginine to form the product NO, which inhibits the function of effector T cells.^10^

Expression of immune checkpoints, such as programmed cell death protein 1(PD‐1), is an important way for TAMs to regulate the tumor‐killing function of T cells.^6^ Several studies have investigated the ability of TAMs to modulate the expression of PD‐1/programmed death‐ligand 1 (PD‐L1) via several cytokines in the breast TME. For example, IFN‐γ secreted by TAMs activates the JAK/STAT3 and PI3K/AKT pathways to upregulate PD‐L1 expression. TGF‐β, a multifunctional cytokine, induces macrophage polarization to M2, thereby enhancing the suppressive activity of TAMs while inducing upregulation of PD‐L1 promoting tumor escape. In the IL‐6 deficient condition, PD‐L1 expression was significantly upregulated, and treatment with an anti‐PD‐L1 antibody proved to be remarkably effective.^14^ Moreover, deficiency of macrophage common lymphatic endothelial and vascular endothelial receptor‐1 **(**CLEVER‐1) markedly impedes tumor development via activation of the tumor‐killing ability in effector T cells.^43^

The TAMs play an important role in cancer disease progression since they can exhaust CD8^+^ T cells, leading them to lose their ability to eliminate cancer cells.^44^, ^45^ Thus, as a potential therapeutic rationale in the development of cancer immunotherapy, it is necessary to elucidate the mechanism by which TAMs cause T cell exhaustion. In the TNBC‐ based study conducted by Xu and colleagues, the interaction between TAMs and exhausted T cells were demonstrated using single‐cell transcriptome analysis. The findings indicated that lymphocyte activating 3 (LAG3) and T cell immunoglobulin and mucin domain‐containing protein 3 (TIM3) were enriched during T cell exhaustion when compared to PD‐1 and CTLA‐4, providing targets for potential immune‐based therapies.^46^ Calcium/calmodulin‐dependent protein kinase kinase (CaMKK2), highly expressed within TAMs in breast cancer, can suppress proliferation and T cell tumor killing function.^47^ Additionally, high cyclooxygenase‐2 (COX‐2) expressing hepatocellular carcinoma cell lines can induce M2 TAMs polarization，which can contribute to the exhaustion of the antitumor abilities in activated CD8+ T cells.^48^ When the number of TAMs in the stroma increases, the cells secrete STAT3 into the TME, causing CD8+T cell exhaustion.^44^ Similarly, Pu et al asserted that TAM‐derived extracellular vesicles (EVs) promoted CD8+ T cell exhaustion in a hepatocellular carcinoma (HCC) mice model. The microRNA‐21‐5p (miR‐21‐5p) expression was upregulated in EVs that were carried into tumor tissues. Inhibition of miR‐21‐5p blocked the tumor‐promoting effect of TAMs.^49^ Another study demonstrated that exosomal microRNA‐146a‐5p (miR‐146a‐5p) from TAMs drives T cell exhaustion in HCC.^50^

TAMs are involved in tumor immune regulation by numerous potential mechanisms. TAMs and myeloid‐derived suppressor cells (MDSCs) exert their immunosuppressive effects in a cell contact–dependent manner. Skewed macrophages which transform into TAMs can be induced by MDSCs, and are characterized by downregulation of IL‐12 expression. TAMs stimulate MDSCs to upregulate IL‐10 expression, resulting in secretion of IL‐12 in macrophages further downregulating, forming the self‐perpetuating negative loop damage effector T cell function.^51^ TAMs blunt the function of effector T cells through secretion of IL‐10 from TAMs inhibits IL‐12 production by dendritic cells, leading to blunting of effector T cell function by TAMs.^52^ TAMs play a critical role in suppressing T cell recruitment; however, the potential mechanism of action is still unknown. Targeting the CSF1/CSF1R pathway can obstruct macrophage recruitment and enhance T cell infiltration during chemotherapy or high‐dose irradiation. Similar results were observed when blocking the CCL2/CCR2 pathway which led to macrophage recruitment.^53^ Additionally, classically activated macrophages can be induced by Th1 cytokines (IFN‐γ and TNF‐α), while alternatively activated macrophages can be induced by Th2 cytokines (IL‐13 and IL‐4).^54^ As previously mentioned, TAMs are involved in the immunosuppression of breast cancer and can protect cancer cells.

## TAMS REGULATE ENERGY METABOLISM IN BREAST CANCER CELLS

TAMs impact the overall metabolic profile of the TME through modulation of metabolic activities and metabolites which can influence tumor development.^6^ A large number of macrophages localize significantly in the hypoxic tumor regions and the lactic acid produced by glycolysis in the cancer cells cause them to polarize into M2 phenotype.^10^ Lactate‐activated macrophages promote the secretion of CCL5 by activating the Notch pathway. CCL5 play a key role in translating the metabolic communication between TAMs and breast cancers, increasing aerobic glycolysis, migration and invasiveness of the cancer cells. Blocking the CCL5‐CCR5 axis with monoclonal antibodies disrupts the glycolytic metabolic cycle and inhibits cancer cell metastasis.^55^ The G protein‐coupled receptor 132 (Gpr132), expressed by TAMs in a high‐lactate environment, is a key sensor of the rising lactate levels in TME, mediated the interaction between cancer cells and TAMs during metastasis. Lactate‐activated Gpr132 is involved in M2‐type macrophage polarization.^56^ TAMs upregulate the expression of HIFs, which assists them in adapting to the hypoxic TME. The HIFs function as important regulatory players modulating the tumor energy metabolism.^21^ HIF‐1α drives glycolytic metabolism in metastatic breast cancer cells and promotes metastasis and colonization of cancer cells to the liver.^57^ Chen and colleagues have demonstrated that RNA is also involved in aerobic glycolysis. TAMs promote aerobic glycolysis in breast cancer via HIF‐1α‐stabilizing long noncoding RNA (HISLA). The lactate released from glycolytic tumor cells further induces expression of HISLA in macrophages, creating a positive feedback loop for glycolysis that enhances drug resistance in cancer cells.^58^ Additionally, HIF‐2α expression activates mitochondrial oxidative phosphorylation in tumor cells and over‐activation of mitochondrial oxidative phosphorylation is a marker for an aggressive form of breast cancer.^59^ Therefore, HIFs are important energy metabolic targets for breast cancer treatment.

## REPOLARIZATION OF TAMS INTO M1 TYPE MACROPHAGES EXERT TUMOR‐KILLING EFFECTS

The altered TME causes the TAMs to polarize into M1 macrophages and mediate an antitumor immune response.^11^ M1 macrophages highly express proinflammatory factors, such as IL‐6, IL‐12, iNOS, reactive oxygen species (ROS), TNF‐α, which can exert effects to kill tumor cells.^8^ M1 macrophages have a stronger antigen‐presenting ability because they express major histocompatibility complex (MHC) class II.^54^ Repolarization of TAMs into M1 macrophages can inhibit tumor progression by exploiting the plasticity of TAMs.^19^ The maintenance of the immunosuppressive phenotype of TAMs is closely related to the NF‐κB signaling pathway. When NF‐κB signaling is inhibited specifically in TAMs, they repolarize to M1 macrophages and abundantly secrete IL‐12. The IL‐12 can activate and recruit NK cells to perform tumor‐killing functions in advanced tumors.^60^ Upregulation of miR‐155 expression levels drives repolarization of TAMs to M1 macrophages to regain tumor‐killing functions.^61^ Paclitaxel converts TAMs into M1 macrophages via the Toll‐like receptor 4 (TLR4) pathway.^62^ Exosomes of M1 macrophages have been reported to enhance the therapeutic effect of paclitaxel in breast cancer through macrophage‐mediated inflammation.^63^ The combination of anti‐CD40 with anti‐CSF‐1R immunotherapy has been reported to prompt the TAMs to polarize towards a proinflammatory phenotype with antitumor functionality, significantly enhancing the antitumor response and prolonging the survival in patients.^64^ A recent study showed that anti‐Her2 antibody alone was able to upregulate PD‐L1 in macrophages which led to immunosuppression and poor prognosis. Interestingly, a combination of therapeutic antibodies and anti‐PD‐L was shown to be beneficial.^65^ Traditional Chinese medicine can promote repolarization of TAMs, and may serve as a novel treatment modality for breast cancer treatment in the future.^66^ An important example of an effective Chinese herbal medicine‐based anticancer agent is emodin, which exerts antitumorigenic effects in breast cancer by inhibiting the TGF‐β1 production in the macrophages which in turn suppresses TAM polarization.^32^, ^67^ Additionally, XIAOPI formula (XPS) is being extensively used as a promising traditional chinese medicine‐based therapy in breast cancer treatment. Baohuoside I (BHS) is the key bioactive compound of XPS. Functional studies have revealed that BHS can suppress the M2 phenotype polarization of TAMs to significantly inhibit the migration and invasion of breast cancer cells.^68^

## FUTURE DEVELOPMENT

One of the main limitations of targeting TAMs for cancer therapy is the lack of reliable and specific markers. Cassetta et al. used multicolor flow cytometric analysis to determine the sialic acid‐binding Ig‐like lectin 1 (SIGLEC1) protein expression in breast cancer patients and found that SIGLEC1 was exclusively expressed by TAMs. Furthermore, in the circulation, both classical and nonclassical monocytes exhibited low expression of SIGLEC1, with no difference between cancer and noncancer patients, indicating the specificity of SIGLEC1 to macrophages/TAMs.^69^ Additionally, breast cancer cells overexpress CD24, while TAMs express high levels of Siglec‐10. Genetic ablation of Siglec‐10 robustly resulted in a macrophage‐dependent reduction of tumor growth.^70^ This study emphasizes the existing knowledge concerning the role of TAMs in breast cancer and attempts to identify unique genes expressed by human TAMs to uncover novel therapeutic targets.

## CONCLUSIONS

This article summarizes the roles played by TAMs in breast cancer development by promoting TME angiogenesis and cancer cell metastasis, inducing cancer cell stemness, regulating energy metabolism, and supporting immune system suppression..Macrophage function and polarization are regulated by multiple TME‐based factors. TAMs are important players in tumor progression which should be explored with the aim of developing improved therapies for breast cancer treatment. Macrophages can adopt different states of activation. Repolarization of TAMs into antitumorigenic M1 macrophages is a very promising therapeutic option. A recent study conducted by Xiao et al. showed the presence of a high proportion of M2‐like TAMs reaching 43.1% in control groups. In comparison, M2‐like TAMs decreased to 10.7% in the treatment group by M2 repolarizing to M1. In addition, the proportion of M1 macrophages increased from 10.2% to 58%,^71^which apparently contributed to the effective inhibition of tumor growth and metastasis with low immune side‐effects. In future, combination treatment modalities involving traditional chemotherapeutic drugs and traditional Chinese medicine targeted at promoting repolarization of TAMs can serve as a novel treatment modality for breast cancer treatment. Therefore, exploring the role and mechanism of action of TAMs in the development of breast cancer can provide a foundation for better treatment of breast cancer.

## CONFLICT OF INTEREST

The authors confirm that there are no conflicts of interest.
