# Corrigendum

**DOI:** 10.1111/1759-7714.14709

**Published:** 2022-12-01

In Xinqun Huang *et a*l.^1^ the following error was published on page 270.

The authors noticed that the Figure 1 in this paper is incorrect. The correct image is shown below:

**FIGURE 1:**
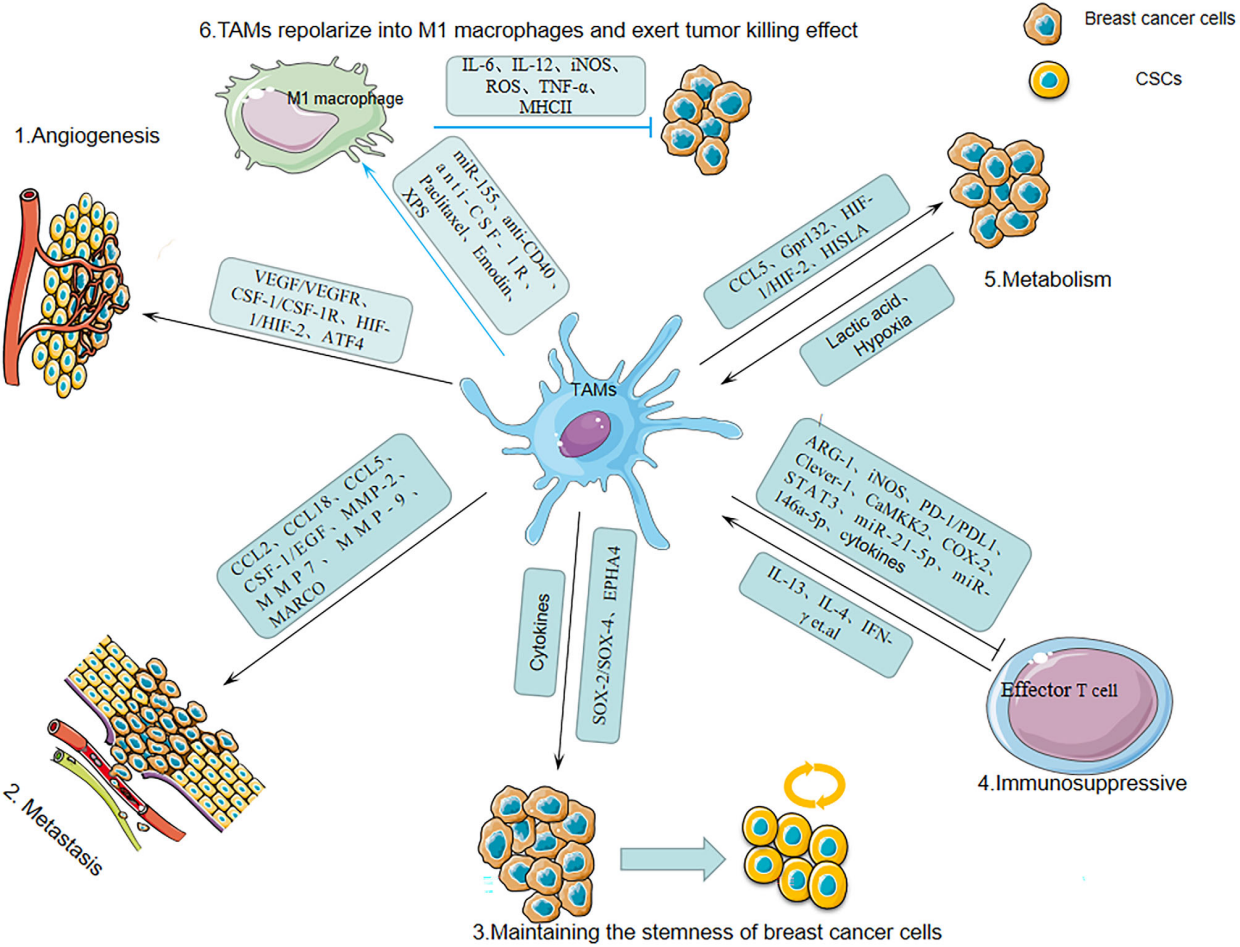
TAM‐associated mechanisms which promote the development of breast cancer

The authors apologize for the error and any inconvenience it may have caused.
